# Analysis of management of non-invasive ventilation support in prehospital care for COPD patients and short-term outcome

**DOI:** 10.1186/cc12082

**Published:** 2013-03-19

**Authors:** J Cuny, G Campagne, P Gosselin, P Goldstein, N Assez, E Wiel

**Affiliations:** 1 Lille University Hospital Center, Lille Cedex, France

## Introduction

Benefits of the use of NIV in emergency departments are well established. Training and available staff, and choice of respiratory machines are essential criteria for success.

## Methods

We conducted an observational, descriptive, retrospective, single-center study in a 4-month period. COPD patients with respiratory failure who received prehospital NIV were included. We compared two groups: COPD patients with NIV, and COPD patients without NIV.

## Results

Forty-two patients were included, mean age 68.86 years (± 11.98), 57.14% smokers, 64.28% arterial hypertension, 100% longterm oxygenotherapy, 23.80% antibiotics in the 7 days before, 28.57% corticosteroids. A total of 88.09% had bronchospasm, 78.26% had struggle signs, 28.57% were unable to speak, 14.28% of patients were sweating. The mean respiratory rate was 30.5 cycles/minute (± 7.17), mean pulse rate was 105.76 (± 25.34). Nasal EtCO2 was 47.75 mmHg (± 16.53), pulse oxymetry in air was 85% (± 10.94), oxygen flow rate was 5.45 l/minute (± 2.42), temperature was 37.14°C (± 8.15). Twenty patients received NIV. A total of 61.90% were admitted to the emergency department, 35.71% to the ICU, and one patient was left at home. One patient received tracheal intubation in the hosting service. Mortality in the first month was 13.04%. A significant difference (*P <*0.05) was found for: sweats (30%/0), respiratory rate (34 ± 8.23/27 ± 6.11), nasal EtCO_2 _(55.0 ± 24.4/40.50 ± 9.03), pulse oxymetry in air (80% ± 8.63/90% ± 13.25), pulse oxymetry with oxygen (89.4% ± 4.24/87.90% ± 2.55), β_2_-mimetic and anti-cholinergic nebulization (60% ± 0.5/90% ± 0.29), emergency room admission (35% ± 0.35/86% ± 0.48), ICU admission (60% ± 0.5/13% ± 0.35), arterial blood gases on arrival in the host service (PaCO_2 _76.6 ± 18.66/43.93 ± 11.78). No difference in mortality at 1 month (2/3).

## Conclusion

Non-invasive ventilation has improved the management and prognosis of COPD patients admitted to the emergency room. Very few studies concern the prehospital management. NIV seems to show an effect on prehospital care, especially in patients with signs of severity, hypercapnia, and without fever. Oxygenation and hypercapnia seem to be improved. Also fewer patients are admitted to the ICU. Bronchospasm does not seem to be an obstacle.
